# Acetate as substrate for  l-malic acid production with *Aspergillus oryzae* DSM 1863

**DOI:** 10.1186/s13068-021-01901-5

**Published:** 2021-02-23

**Authors:** Aline Kövilein, Julia Umpfenbach, Katrin Ochsenreither

**Affiliations:** Institute of Process Engineering in Life Sciences 2 – Technical Biology, Karlsruhe Institute of Technology (KIT), Fritz-Haber-Weg 4, 76131 Karlsruhe, Germany

**Keywords:** Malate, Acetic acid, Dicarboxylic acid, Organic acid, Fermentation, Bioprocess, Carbon source, Filamentous fungus, Microbial production of bio-based chemicals, Calcium carbonate

## Abstract

**Background:**

Microbial malic acid production is currently not able to compete economically with well-established chemical processes using fossil resources. The utilization of inexpensive biomass-based substrates containing acetate could decrease production costs and promote the development of microbial processes. Acetate is a by-product in lignocellulosic hydrolysates and fast pyrolysis products or can be synthesized by acetogens during syngas fermentation. For the fermentation of these substrates, a robust microorganism with a high tolerance for biomass-derived inhibitors is required. *Aspergillus oryzae* is a suitable candidate due to its high tolerance and broad substrate spectrum. To pave the path towards microbial malic acid production, the potential of acetate as a carbon source for *A. oryzae* is evaluated in this study.

**Results:**

A broad acetate concentration range was tested both for growth and malic acid production with *A. oryzae*. Dry biomass concentration was highest for acetic acid concentrations of 40–55 g/L reaching values of about 1.1 g/L within 48 h. Morphological changes were observed depending on the acetate concentration, yielding a pellet-like morphology with low and a filamentous structure with high substrate concentrations. For malic acid production, 45 g/L acetic acid was ideal, resulting in a product concentration of 8.44 ± 0.42 g/L after 192 h. The addition of 5–15 g/L glucose to acetate medium proved beneficial by lowering the time point of maximum productivity and increasing malic acid yield. The side product spectrum of cultures with acetate, glucose, and cultures containing both substrates was compared, showing differences especially in the amount of oxalic, succinic, and citric acid produced. Furthermore, the presence of CaCO_3_, a pH regulator used for malate production with glucose, was found to be crucial also for malic acid production with acetate.

**Conclusions:**

This study evaluates relevant aspects of malic acid production with *A. oryzae* using acetate as carbon source and demonstrates that it is a suitable substrate for biomass formation and acid synthesis. The insights provided here will be useful to further microbial malic acid production using renewable substrates.

## Background

In view of climate change and the depletion of fossil resources, the microbial production of chemicals currently synthesized from crude oil, gas or coal is of great interest. A key factor for the replacement of chemical processes is the economic viability of the respective bioprocesses. As substrate costs are a large part of the overall costs for microbial processes, the utilization of low-priced substrates is essential [1, 2]. Acetate is produced in various processes based on biomass including the hydrolysis of lignocellulose, syngas fermentation and fast pyrolysis [3–5]. It could therefore represent a promising low-cost carbon source for the renewable production of widely used chemicals.

Malic acid, a C4-dicarboxylic acid, is one of the commercially used chemicals which is still produced from fossil resources. Its main route of synthesis is via the transformation of maleic anhydride derived from petroleum or natural gas. Malic acid production is on the rise with a current global production volume of about 80,000–100,000 ton/year [6]. While the majority is used as acidulant and taste enhancer in the food industry, further areas of application encompass its usage in personal care, pharmaceutical and cleaning formulations, as animal feed additive and in the production of polymers. In view of an integrated bioeconomy, malic acid could replace maleic anhydride as building block chemical for the synthesis of various products including succinic anhydride, 1,4-butanediol, tetrahydrofuran and γ-butyrolactone [6, 7].

As intermediate of the tricarboxylic acid (TCA) cycle, malic acid has great potential to be produced microbially. Filamentous fungi including species of *Aspergillus*, *Ustilago* and *Rhizopus* have been identified as efficient natural malic acid producers [8]. Among these genera, *Aspergillus oryzae* is of particular interest in the context of malic acid production with bio-based substrates as this species exhibits a high tolerance against biomass-derived inhibitors [9]. The majority of malic acid production processes with *A. oryzae* described so far utilized glucose as carbon source. With this substrate, malic acid titers between 58 and 165 g/L, yields of 0.76–1.38 mol/mol and productivities up to 1.38 g/(L*h) were reported [10–13]. However, glucose is an edible sugar and its production takes up valuable cropland. The substrate spectrum of *A. oryzae* DSM 1863 also encompasses xylose and glycerol which yielded 39.4 g/L and 45.4 g/L malic acid, respectively, while the production of about 38 g/L malic acid was reported with a hydrolysate of beech wood cellulose [10, 14]. Oswald et al. were the first to demonstrate that malic acid production is also possible using acetate derived from syngas fermentation [15]. However, the authors only obtained very low malic acid concentrations of 1.8 g/L, 1.4 g/L and 0 g/L in a triplicate fermentation performed in a 2-L stirred-tank reactor, elucidating the need for process improvement. Besides the work of Oswald et al., acetate has not been further explored as substrate for malic acid production with *A. oryzae* to the authors’ best knowledge.

To render malic acid production with acetate more efficient and further the utilization of inexpensive acetate-containing substrates, this work evaluates important aspects of malic acid production with acetate as carbon source. A broad range of acetate concentrations was tested for both growth and acid production to find the optimum for each stage. Furthermore, the influence of adding low amounts of glucose to acetate medium was evaluated. Additionally, the side product spectrum of *A. oryzae* depending on the substrate type and concentration was identified and the necessity of adding CaCO_3_ for efficient acid production was demonstrated.

## Results

### Acetate as substrate for growth of *A. oryzae*

To determine the optimum acetate concentration for biomass production with *A. oryzae*, substrate concentrations of 5–70 g/L were evaluated. Fungal growth was quantified by dry biomass concentration as well as substrate and ammonium consumption after 48 h of incubation. As presented in Table 1, biomass formation was detected for all substrate concentrations. The highest dry biomass titers were obtained with acetic acid concentrations of 40–55 g/L with values ranging between 1.08 ± 0.14 g/L and 1.14 ± 0.11 g/L. Below 40 g/L and above 55 g/L the dry biomass concentration decreased and the lowest concentration of 0.12 ± 0.02 g/L was measured for cultures with 70 g/L acetic acid. The substrate and ammonium consumption showed a correlating behavior. The highest consumption values were obtained with acetic acid concentrations between 40 and 55 g/L in the range of 3.47 ± 0.19 g/L to 5.68 ± 0.31 g/L for acetate and 0.17 ± 0.06 g/L to 0.22 ± 0.03 g/L for ammonium. The overall substrate consumption within 48 h was low as no more than 16% of the available acetate (cultures with 10 g/L acetic acid) was consumed. Regarding the ammonium consumption, the highest proportion of 20% ammonium (initial ammonium concentration of 1.09 g/L) was observed for cultures with 55 g/L acetic acid. The biomass yields decreased with increasing substrate concentration and ranged between 0.17 ± 0.08 g/g (70 g/L acetic acid) and 0.76 ± 0.19 g/g (5 g/L acetic acid).

**Table 1:** Comparison of growth of *A. oryzae* with different acetic acid concentrations after 48 h

c (acetic acid) (g/L)	Consumed substrate (g/L)	Consumed ammonium (g/L)	c (dry biomass) (g/L)	Y_X/S_ (g/g)
5	0.76 ± 0.14	0.05 ± 0.02	0.56 ± 0.09	0.76 ± 0.19
10	1.54 ± 0.24	0.16 ± 0.03	0.79 ± 0.08	0.52 ± 0.05
20	1.65 ± 0.11	0.16 ± 0.02	0.80 ± 0.10	0.48 ± 0.03
30	2.05 ± 1.09	0.15 ± 0.07	0.92 ± 0.11	0.54 ± 0.29
40	3.47 ± 0.19	0.18 ± 0.04	1.14 ± 0.11	0.33 ± 0.04
45	5.68 ± 0.31	0.21 ± 0.04	1.11 ± 0.10	0.18 ± 0.03
50	3.48 ± 0.42	0.17 ± 0.06	1.11 ± 0.15	0.33 ± 0.09
55	4.25 ± 0.49	0.22 ± 0.03	1.08 ± 0.14	0.25 ± 0.03
60	2.86 ± 0.67	0.15 ± 0.03	0.74 ± 0.12	0.26 ± 0.04
65	1.97 ± 0.54	0.12 ± 0.01	0.53 ± 0.11	0.27 ± 0.02
70	0.55 ± 0.56	0.03 ± 0.02	0.12 ± 0.02	0.17 ± 0.08

Y_X/S_ = substrate specific dry biomass yield calculated as g(dry biomass)/g(consumed acetic acid)

**Fig. 1:**
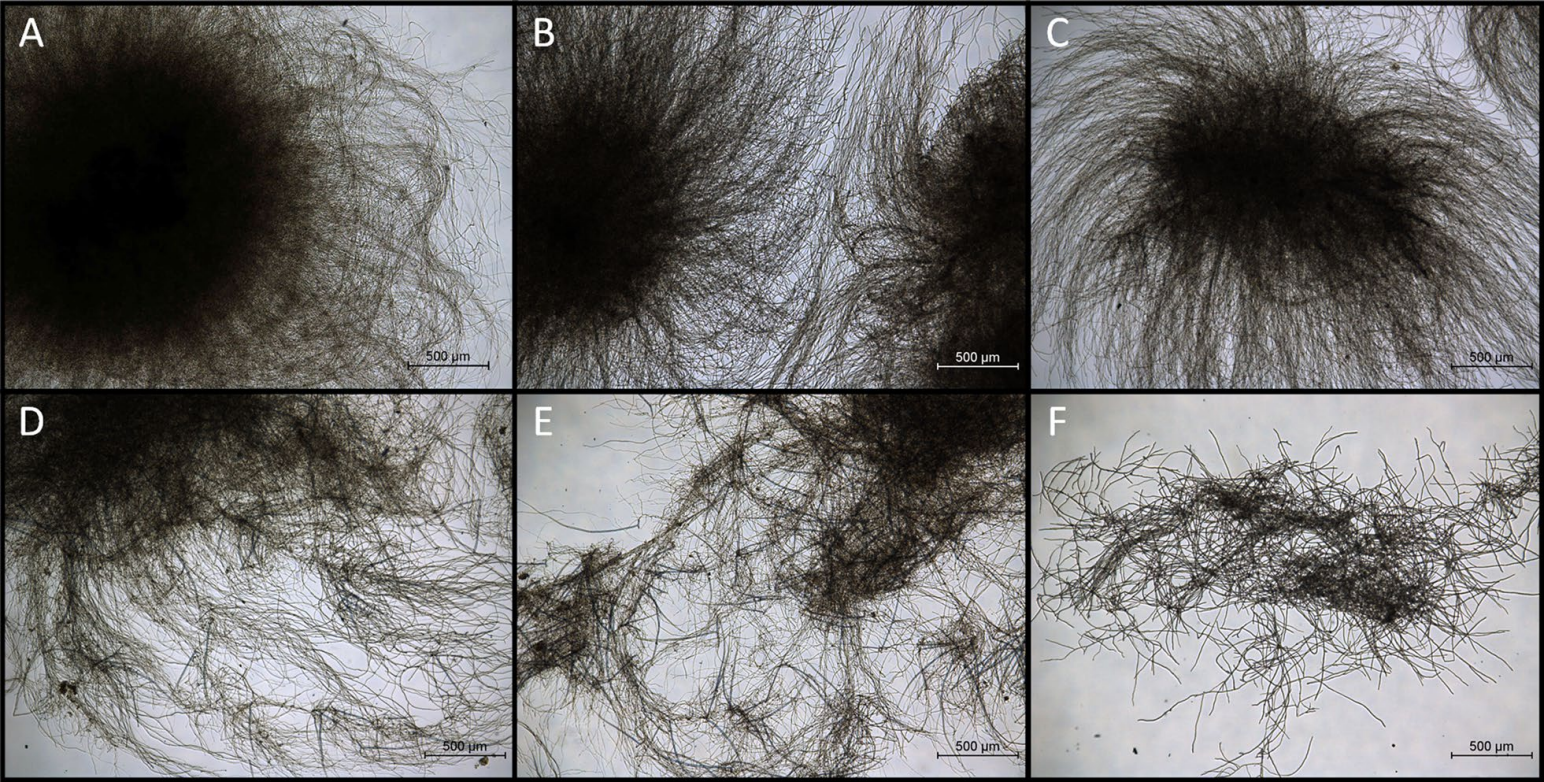
Morphology of *A. oryzae* after 48 h of growth with 5 g/L (**a**), 20 g/L (**b**), 30 g/L (**c**), 45 g/L (**d**), 55 g/L (**e**) and 65 g/L (**f**) acetic acid. Cultures were incubated at 30 °C and 100 rpm

The morphology of *A. oryzae* was strongly influenced by the concentration of acetate in the medium as depicted in Fig. 1. Microscopic analysis showed a pellet-like morphology with acetic acid concentrations up to 30 g/L while the size of the pellet core decreased with increasing acetate concentration. With a substrate concentration of 5 g/L acetic acid, the pellet core size was about 2 mm while it was only about 1.0–1.5 mm with 30 g/L acetic acid. Concentrations above 30 g/L acetic acid led to a highly branched filamentous structure and no pellets were formed.

### Acetate as substrate for malic acid production with *A. oryzae*

**Fig. 2:**
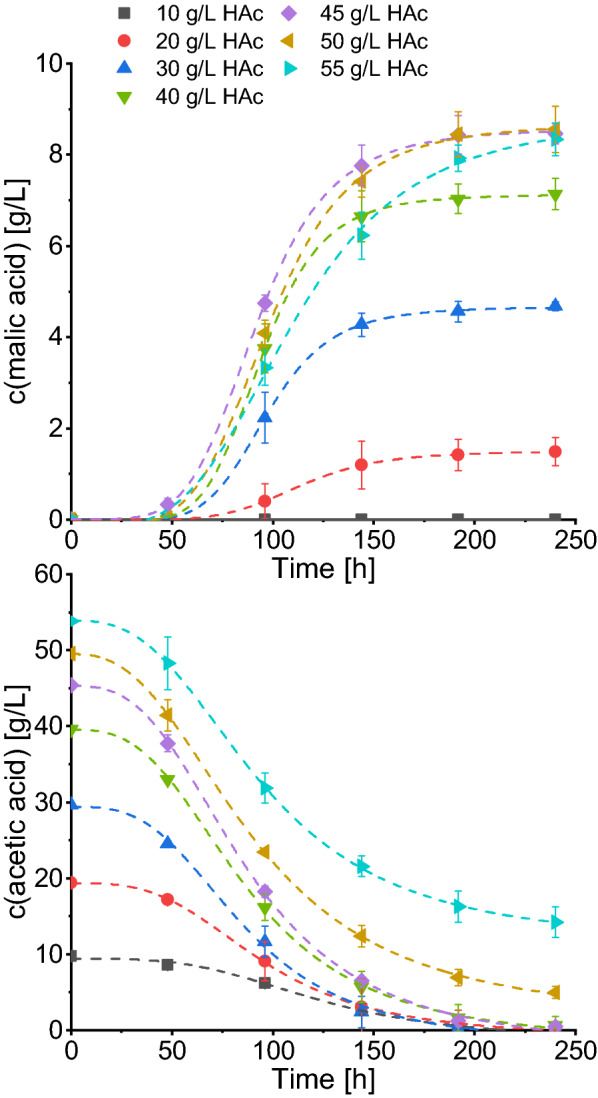
Comparison of malic acid production with initial acetic acid concentrations of 10–55 g/L. Cultures were incubated at 32 °C, 120 rpm in the presence of 90 g/L CaCO_3_. Acetate consumption and acid production was fitted using Eq. 1. Results are the mean of triplicate experiments and error bars indicate the standard deviation. *HAc  *acetic acid

Based on the results of the growth experiments, acetic acid concentrations between 10 and 55 g/L were evaluated for malic acid production with *A. oryzae*. In the acid production stage, the ammonium concentration was reduced to 0.33 g/L and 90 g/L CaCO_3_ was added. The pH of all main culture media was set to 5.5, thus lower than that of the pre-culture media (pH 6.5) to level out the pH increase caused by the addition of CaCO_3_. In preliminary experiments, initial medium pH values of 4.5–6.5 were tested with 45 g/L acetic acid and the highest malic acid production was observed for an initial medium pH of 5.5 (Additional file 1: Figure S1). Concentration curves for malic and acetic acid are displayed in Fig. 2 and calculated values are summarized in Table 2. Acetate consumption and acid production showed a characteristic logistic behavior. After 48 h, malic acid was first detected in cultures with 45 g/L acetic acid (0.33 ± 0.13 g/L). The majority of malic acid production was completed within 144 h and only a slight further increase was monitored between 144 and 192 h. After 192 h, no significant increase of malic acid concentration was observed. For this reason, all calculations of Table 2 are based on the measurements at 192 h. The highest malic acid concentrations after 192 h were observed with 45 g/L (8.44 ± 0.42 g/L) and 50 g/L acetic acid (8.45 ± 0.49 g/L). With decreasing substrate concentration, lower malic acid titers were obtained. While with 20 g/L acetic acid 1.42 ± 0.34 g/L malic acid was produced, no acid production was observed in cultures with 10 g/L acetic acid. For the cultures with 10–45 g/L initial acetic acid, the carbon source was either depleted or very close to depletion after 192 h whereas for the two highest substrate concentrations still about 14% (50 g/L acetic acid) and 30% (55 g/L acetic acid) of the acetic acid remained. Maximum productivities and respective time points were calculated as the maximum of the first derivative of the fitted curve (Eq. 1). Maximum productivities ranged between 0.020 g/(L*h) (20 g/L acetic acid) and 0.123 g/(L*h) (40 g/L acetic acid). The time point of maximum productivity was lowest for cultures with 45 g/L acetic acid (85 h) and highest using 20 g/L acetic acid (107 h). As for the overall productivities, the highest value of 0.044 g/(L*h) was obtained for cultures with 45 and 50 g/L acetic acid. Regarding the nitrogen consumption, no ammonium could be detected after 96 h of cultivation with acetic acid concentration of 30–55 g/L while with 20 g/L acetic acid about 5% of the nitrogen was left. In the cultures with 10 g/L acetic acid nitrogen limitation was only reached after 192 h (Additional file 1: Figure S2).

**Table 2:** Comparison of malic acid production with *A. oryzae* and different substrate concentrations after 192 h

Substrate	c (substrate) (g/L)	Consumed substrate (g/L)	c (malic acid) (g/L)	Y_P/S_ (g/g)	Y_P/S, carbon_ (g/g)	Overall Productivity (g/(L*h))	Maximum productivity (g/(L*h))	Time of maximum productivity (h)
HAc	10	9.72 ± 0.15	0.00 ± 0.00	0.00	0.00	0.000	0.000	0.00
	20	18.43 ± 1.54	1.42 ± 0.34	0.08	0.07	0.007	0.020	107.15
	30	29.64 ± 0.06	4.56 ± 0.23	0.15	0.14	0.024	0.076	92.25
	40	38.01 ± 1.74	7.04 ± 0.33	0.19	0.17	0.037	0.123	89.61
	45	44.14 ± 0.53	8.44 ± 0.42	0.19	0.17	0.044	0.122	85.05
	50	42.58 ± 0.80	8.45 ± 0.49	0.20	0.18	0.044	0.115	90.33
	55	37.55 ± 2.27	7.92 ± 0.29	0.21	0.19	0.041	0.081	95.38
Mixed	HAc: 45 glucose: 5	HAc: 44.87 ± 0.30 glucose: 5.06 ± 0.04	9.95 ± 0.25	0.20	0.18	0.052	0.114	61.98
	HAc: 45 glucose: 10	HAc: 44.46 ± 0.30 glucose: 10.07 ± 0.03	12.15 ± 0.21	0.22	0.20	0.063	0.119	58.62
	HAc: 45 glucose: 15	HAc: 44.66 ± 0.33 glucose: 15.06 ± 0.04	14.18 ± 0.09	0.24	0.21	0.074	0.138	61.02
Glucose^a^	109	58.90 ± 0.86	38.75 ± 0.29	0.66	0.59	0.231	0.292	87.61

^a^ The values for 109 g/L glucose were determined for a cultivation time of 168 h

*HAc*  acetic acid, *Y*_*P/S*_* (g/g)  * substrate specific malic acid yield calculated as g(malic acid)/g(total consumed substrate(s)), *Y*_*P/S, carbon*_* (g/g)  *substrate specific malic acid yield calculated as g(produced C from malic acid)/g(total consumed C from substrate(s))

**Fig. 3:**
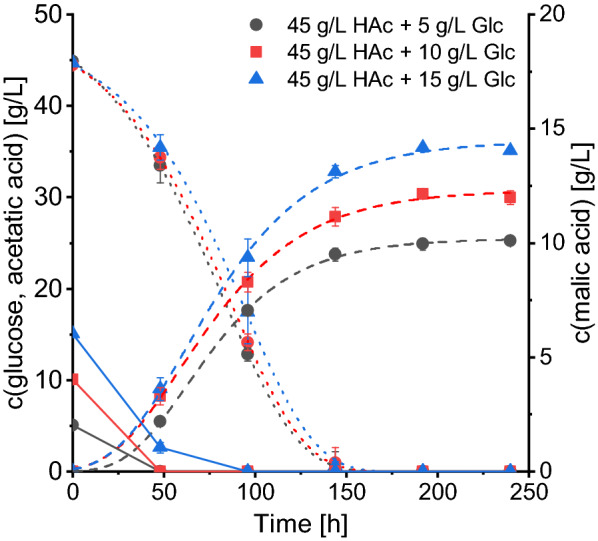
Malic acid production with 45 g/L acetic acid and 5–15 g/L glucose. Acetic acid: dotted lines, malic acid: dashed lines, glucose: solid lines. Cultures were incubated at 32 °C, 120 rpm in the presence of 90 g/L CaCO_3_. Acetate consumption and acid production was fitted using Eq. 2. Results are the mean of triplicate experiments and error bars indicate the standard deviation. *HAc*  acetic acid, *Glc* glucose

Besides acetate as sole carbon source, mixtures of 45 g/L acetic acid with 5, 10 and 15 g/L glucose were evaluated for malic acid production. By adding low concentrations of glucose to a medium with acetate as the main carbon source, the possibility of accelerating the production should be assessed. As displayed in Fig. 3, glucose was depleted within 48 h for cultures with 5 and 10 g/L and within 96 h for an initial concentration of 15 g/L of the substrate. In the cultures with 15 g/L glucose about 12 g/L of the glucose was consumed during the first 48 h while at the same time about 10 g/L acetic acid was consumed, illustrating a simultaneous consumption of the two carbon sources. Acetic acid was very close to depletion after 144 h which is faster compared to the cultures using it as sole carbon source in which still about 7 g/L acetic acid remained after 144 h. A maximum malic acid concentration of 14.18 ± 0.09 g/L was quantified using 45 g/L acetic acid + 15 g/L glucose which corresponds to a yield of 0.24 g/g total substrate and a carbon yield of 0.21 g/g. An increase in overall and maximum productivities as well as a decrease in the time of maximum productivity was associated with the presence of glucose in the medium. The lowest time point of maximum productivity (59 h) was calculated for cultures with an addition of 10 g/L glucose which represents a decrease of 31% compared to the cultures with 45 g/L acetic acid only. Regarding the nitrogen consumption, ammonium was consumed faster for all cultures with glucose addition and was depleted after 48 h (Additional file 1: Figure S2).

Table 2 furthermore displays values for the malic acid production process based on glucose. As the process with glucose is faster than the one with acetate, all calculations were performed with the data obtained after 168 h of cultivation. With an initial glucose concentration of 109 g/L, 38.75 ± 0.29 g/L malic acid was produced, corresponding to a yield of 0.66 g/g and an overall productivity of 0.231 g/(L*h).

The pH value in the cultures with acetate showed a strong increase until the end of the cultivation time. After 192 h, the pH was between 9.5 and 9.8 for all tested acetic acid concentrations and for the cultures with mixed substrate. With glucose, by contrast, the pH decreased to a value of about 6.6 within 168 h (Additional file 1: Figure S3).

### Side products of malic acid production with *A. oryzae*

During the acid production stage, not only malic acid but also several other organic acids were produced by *A. oryzae*. All side products for the cultures with 10–55 g/L acetic acid, 45 g/L acetic acid plus 5–15 g/L glucose, and 109 g/L glucose were quantified and are summarized in Table 3. The concentration curves for all acids were similar to the one of malic acid, starting to increase between 48 and 96 h and increasing until about 192 h and are therefore not displayed individually. As indicated in the table, the acid composition was influenced by the substrate type and concentration.

**Table 3:** Organic acid distribution for cultivation of *A. oryzae* with different substrate concentrations after 192 h

Substrate	c (substrate) (g/L)	c (total acids) (g/L)	Organic acid distribution (%)						
			Malate	Succinate	Fumarate	Pyruvate	α-Ketoglutarate	Oxalate	Citrate
HAc	10	0.58 ± 0.14	0.0	0.0	1.4	0.0	0.0	98.6	0.0
	20	3.95 ± 0.54	35.6	23.3	2.5	0.6	0.9	37.1	0.0
	30	8.37 ± 0.20	54.5	35.0	3.4	0.6	0.6	5.8	0.0
	40	12.69 ± 0.82	55.5	37.1	4.1	0.6	0.5	1.2	1.0
	45	14.86 ± 0.77	56.8	35.9	4.2	0.8	0.4	0.8	1.1
	50	15.11 ± 0.92	55.9	37.1	4.2	0.6	0.5	0.7	1.0
	55	14.32 ± 0.32	55.3	37.1	4.7	0.6	0.4	0.9	0.9
Mixed	HAc: 45 glucose: 5	20.54 ± 0.42	48.5	41.5	5.3	2.7	0.3	0.4	1.4
	HAc: 45 glucose: 10	25.28 ± 0.39	48.1	39.3	5.9	4.8	0.3	0.4	1.2
	HAc: 45 glucose: 15	29.75 ± 0.23	47.7	39.7	5.9	4.9	0.3	0.2	1.3
Glucose^a^	109	51.88 ± 1.15	74.7	14.6	1.5	0.4	0.3	0.3	8.1

^a^ The values for 109 g/L glucose were determined for a cultivation time of 168 h

*HAc*   acetic acid; percentages were calculated based on the acid concentration in g/L as fraction of the total acid concentration in g/L

For the cultures with acetate as sole carbon source, the total acid concentration increased from 0.58 ± 0.14 g/L for cultures with 10 g/L acetic acid to 15.11 ± 0.92 g/L with 50 g/L acetic acid. With an acetic acid concentration of 55 g/L the total acid concentration decreased again to a value of 14.32 ± 0.32 g/L. Interestingly, acid production was also detected in cultures with 10 g/L acetic acid for which no malic acid could be quantified as described in the previous section. With this substrate concentration, oxalic acid was the main product representing about 99% of the total acid concentration. The oxalic acid percentage decreased with increasing substrate concentration and was lowest for cultures with 50 g/L acetic acid (0.7%). The highest malic acid proportion was quantified with 45 g/L acetic acid (56.8%). Succinic acid represented the main side product for acetic acid concentrations of 30 g/L and above with values between 35.0% and 37.1%. While for an acetate concentration of 30 g/L, oxalic acid was the third largest by-product, this position was taken by fumaric acid for 40–55 g/L acetic acid with values between 4.1% and 4.7%. Citric acid was only determined with acetic acid concentrations of 40–55 g/L and did not exceed 1.1%. Pyruvic acid and α-ketoglutaric acid were produced in minor concentrations below 1.0% of the totally produced acid.

Regarding the mixtures of 45 g/L acetic acid and glucose, the total acid concentration reached values between 20.54 ± 0.42 g/L with 5 g/L glucose and 29.75 ± 0.23 g/L with 15 g/L glucose which is a doubling of the concentration compared to cultures without glucose. Malic acid also represented the main product followed by succinic acid as the major side product. However, the distribution of the proportions shifted to the disadvantage of malic acid compared to the cultures with 45 g/L acetic acid only. The percentage of malic acid was about 9% lower while that of succinic acid was up to 6% higher. Furthermore, the addition of glucose was accompanied by an increased secretion of pyruvic acid which represented 4.9% of the total acid production for cultures with 15 g/L glucose compared to 0.8% when glucose was absent. The percentage of oxalic acid, on the other hand, was decreased and accounted for only 0.2–0.4% of the total acids.

Regarding the cultures with 109 g/L glucose, a total acid concentration of 51.88 ± 1.15 g/L was obtained. With 74.7% malic acid and 14.6% succinic acid, the ratio between the two products was clearly shifted to the side of malic acid compared to the cultures containing acetate. With glucose, however, more citric acid was produced which represented 8.1% of the total acid yield. Oxalic, fumaric, pyruvic and α-ketoglutaric acid were produced in minor amounts which did not exceed 1.5%.

### Influence of CaCO_3_ on malic acid production with acetate

**Fig. 4:**
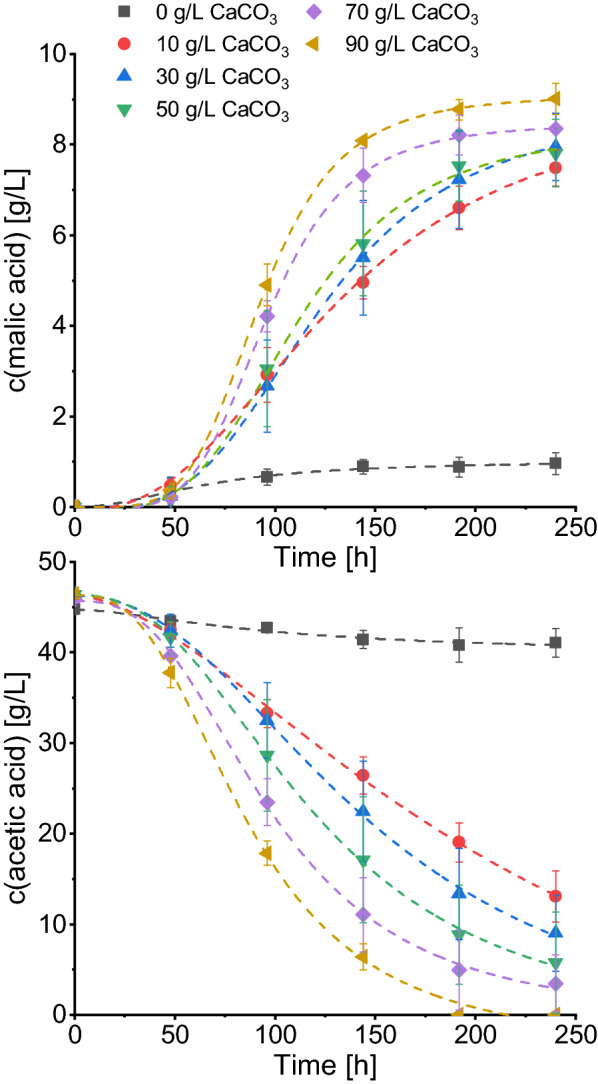
Evaluation of malic acid production with different CaCO_3_ concentrations. Cultures were incubated at 32 °C, 120 rpm with 45 g/L acetic acid. Acetate consumption and acid production was fitted using Eq. 1. Results are the mean of triplicate experiments and error bars indicate the standard deviation

For malic acid production using glucose as carbon source the presence of high CaCO_3_ concentrations has proven necessary for efficient acid synthesis [16]. With the processes of glucose and acetate metabolization being different, it was of interest to elucidate the relationship between the availability of CaCO_3_ and malic acid production with acetate as substrate. Therefore, CaCO_3_ was added to the medium in a range of 0–90 g/L and acid production, substrate and ammonium consumption, and the pH development was monitored. As displayed in Fig. 4 and Table 4, a relationship between malic acid production and the CaCO_3_ concentration was observed. The utilization of 90 g/L CaCO_3_ resulted in the highest malic acid titer of 8.77 ± 0.23 g/L which corresponds to a product yield of 0.19 g/g. Furthermore, the highest maximum productivity of 0.121 g/(L*h) at the lowest corresponding time point of about 85 h was calculated for the highest CaCO_3_ concentration. The utilization of decreasing CaCO_3_ concentrations, however, resulted in reduced malic acid titers, substrate consumptions, and productivities. While with 10 g/L CaCO_3_ still 6.60 ± 0.48 g/L malic acid was detected after 192 h, the titer dropped to 0.88 ± 0.22 g/L when CaCO_3_ was absent. Furthermore, the substrate consumption was greatly reduced. In the cultures with 10 g/L CaCO_3_ 41% of the substrate was left after 192 h while without CaCO_3_ addition even 91% remained. With 90 g/L CaCO_3_, by contrast, all substrate was consumed within this period. This observation clearly illustrates the necessity of high CaCO_3_ concentrations for malic acid production using acetate.

**Table 4:** Comparison of malic acid production with *A. oryzae* and different CaCO_3_ concentrations after 192 h

c (CaCO_3_) (g/L)	Consumed substrate (g/L)	c (malic acid) (g/L)	Y_P/S_ (g/g)	Y_P/S, carbon_ (g/g)	Overall productivity (g/(L*h))	Maximum productivity (g/(L*h))	Time of maximum productivity (h)
0	3.95 ± 2.15	0.88 ± 0.22	0.28	0.25	0.005	0.010	41.32
10	27.07 ± 1.95	6.60 ± 0.48	0.24	0.22	0.034	0.052	95.14
30	33.01 ± 5.07	7.22 ± 1.08	0.22	0.20	0.038	0.067	102.82
50	37.57 ± 5.46	7.54 ± 0.78	0.20	0.18	0.037	0.075	96.58
70	40.82 ± 4.19	8.21 ± 0.44	0.20	0.18	0.043	0.109	87.93
90	46.40 ± 0.15	8.77 ± 0.23	0.19	0.17	0.046	0.121	84.80

*Y*_*P/S*_* (g/g)  *substrate specific malic acid yield calculated as g(malic acid)/g(consumed acetic acid), *Y*_*P/S, carbon*_* (g/g)  *substrate specific malic acid yield calculated as g(produced C from malic acid)/g(consumed C from acetic acid)

The distribution of the side products did not change considerably depending on the CaCO_3_ concentration and was not displayed for this reason. Regarding the pH values, a similar progression was observed for all CaCO_3_ concentrations with the starting values located between 6.4 and 6.7 and final values of 9.4–9.9 (Additional file 1: Figure S4). The nitrogen source was depleted after 96 h of incubation for cultures with 70 and 90 g/L CaCO_3_ and after 144 h with 10–50 g/L CaCO_3_. Without CaCO_3_ about 40% of the initial ammonium was still detected after 240 h (Additional file 1: Figure S5).

## Discussion

*Aspergillus oryzae* is able to metabolize a large variety of substrates and has proven as very robust microorganism which makes it an interesting candidate for the application in processes employing bio-based substrates. Here, we wanted to explore acetate as alternative carbon source by evaluating the growth behavior and several characteristics regarding the malic acid production with this substrate.

### Influence of acetate concentration on morphology and biomass formation

In the pre-culture, the growth behavior of *A. oryzae* up to a substrate concentration of 70 g/L acetic acid was evaluated. While the presence of 40–55 g/L acetic acid yielded similar dry biomass concentrations, lower substrate amounts resulted in reduced growth although only a small proportion of the substrate was consumed. Hence, a substrate limitation cannot explain this observation. The reason for the reduced growth with acetic acid concentrations of 30 g/L and below is most likely the pellet-like morphology of *A.* *oryzae.* Filamentous microorganisms are known to display a high variation in morphological phenotypes ranging from dispersed mycelia to spherical particles termed “pellets”. The morphology is influenced by different cultivation parameters including the temperature, pH value, medium composition, inoculum concentration or mechanical stress [17]. A pellet morphology is advantageous regarding the flow behavior of the culture broth, resulting in a Newtonian flow accompanied by a lower viscosity compared to media containing dispersed mycelia [18]. However, the oxygen and nutrient supply is limited within biomass pellets which can lead to cell lysis in the pellet core. Experiments with *A. niger* showed that in dense pellets with a radius of about 600 µm, the oxygen supply declines to zero within the outer 200 µm of the pellet [19]. The largest pellet cores in the study presented here were obtained with 5 g/L acetic acid, presumably explaining the low biomass production for this substrate concentration. An increase in the acetate amount is accompanied by a rise of the osmotic pressure which is probably the reason for the decreasing pellet size until reaching a mycelial morphology for acetic acid concentrations of 40 g/L and above [20].

The reduced biomass formation observed with acetic acid concentrations above 55 g/L likely originates from an increased concentration of the undissociated acetic acid. The toxicity of acetic acid can be explained by the “weak-acid theory”. According to this theory, the undissociated acid is lipid soluble and thus able to diffuse into the cytosol. It then dissociates due to the higher cytosolic pH which has been reported to be around 7.0 and 7.5 for fungal mycelia [21, 22]. The resulting anions and protons are not able to dissociate back and therefore accumulate in the cytoplasm. The proton release causes a cytosolic pH drop which inhibits enzyme activity [22]. The medium pH for all tested acetic acid concentrations in the pre-culture was set to pH 6.5. As the pKa for acetic acid is 4.76, only a small portion of the substrate is present in its protonated form at this pH value. However, with an increasing acetic acid concentration, the amount of undissociated acid rises. With 45 g/L acetic acid, about 0.8 g/L (0.013 mol/L) are present in the protonated form. For 70 g/L acetic acid, this fraction increases to 1.3 g/L (0.021 mol/L). Alcano et al. showed that spore germination stops when the undissociated acetic acid concentration reaches values between 0.018 and 0.022 mol/L for *Aspergillus flavus*, *Aspergillus carbonarius* and *Aspergillus parasiticus* [23]. Thus, with acetic acid concentrations above 55 g/L and a pH of 6.5, the amount of the protonated acid becomes inhibiting for *A. oryzae*. Another possible inhibition factor in fermentations with high acetic acid concentrations is the increased salt concentration accompanying the adjustment of the medium pH. The amount of NaOH required for the pH adjustment with 45 g/L acetic acid to a value of 6.5 is about 0.72 mol/L while it amounts to 1.12 mol/L for media with 70 g/L acetic acid. Other studies found that the addition of low NaCl concentrations was beneficial for the growth of *Aspergillus* species, whereas high concentrations of 1 mol/L and above inhibited it [24, 25]. As consequence, increased concentrations of sodium and acetate ions present with initial acetic acid concentrations above 55 g/L could have contributed to the reduced biomass formation.

The overall consumption of ammonium during the growth phase was low. Investigating the possibility of a nitrogen reduction in the pre-culture to save process costs should therefore be considered. Evaluating further nitrogen sources besides ammonium sulfate could be interesting to enhance biomass formation and reduce the process time. In our experiments, germination in acetate media only started between 8 and 24 h. As demonstrated for *A. niger*, L-amino acids and complex nitrogen sources such as yeast extract and peptone were better suited to initiate germination than ammonium [26].

### Influence of substrate type and concentration on malic acid production

For an efficient malic acid production with *A. oryzae* using glucose as substrate a high C:N ratio is crucial [10]. The objective thus was to identify the highest applicable acetate concentration before observing an inhibition of malic acid synthesis associated with increased acetic acid concentrations. The highest malic acid titers were obtained with 45 g/L and 50 g/L acetic acid. Since a slightly higher productivity, a lower time point of maximum productivity and substrate depletion after 192 h was observed with 45 g/L acetic acid, this concentration is optimum for malic acid production with *A. oryzae*. In the cultures with 55 g/L substrate, malic acid formation seemed to be inhibited. This is pointed out by a slower acid production and the finding that about a third of the substrate remained unused. Thus, malic acid production of *A. oryzae* is inhibited at lower acetic acid concentrations than biomass formation. Malic acid production diminished with acetic acid concentrations below 45 g/L and was absent in cultures with 10 g/L acetic acid. With the lowest carbon source concentration, ammonium was detected as long as acetic acid was present in the medium. If nitrogen is available, biomass can be produced. It is therefore assumed that most of the substrate was channeled towards biomass formation instead of malic acid production. Hence, malic acid synthesis was most likely not observed due to substrate limitation.

The maximum titer obtained with acetate amounted to only 22% of the concentration produced with glucose. With glucose, a much higher initial carbon concentration can be applied in a batch process, whereas the initial concentration with acetate is limited due to inhibiting effects. Considering a fed-batch process or a continuous process with acetic acid as pH regulator and substrate feed might be the key for higher malate concentrations. Continuous feeding of acetic acid was also successfully applied in other processes like for the cultivation of oleaginous yeasts to obtain a high lipid content [27] and should be evaluated for malic acid production.

Considering that efficient malic acid production with acetate only started after 48 h of cultivation, the possibility of adding low amounts of glucose to shorten the lag phase was evaluated. Indeed, the addition of low glucose concentrations has proven beneficial by decreasing the time of maximum productivity and accelerating the acetate consumption. *A. oryzae* metabolized glucose and acetate simultaneously, suggesting that the acetate metabolism is not suppressed when small amounts of glucose are present. Similar observations were made by Armitt et al. who reported that the enzymes involved in acetate metabolism in *A. nidulans* were as active in mixtures of glucose and acetate as with acetate alone and that acetate was even the preferred carbon source [28]. It must be mentioned that these authors tested only low acetate and glucose concentrations of 0.1 and 0.02 mol/L, respectively. De Lucas et al. used a medium with 0.1 mol/L acetate and added 3% (w/v) glucose and observed that isocitrate lyase activity, a key enzyme involved in acetate metabolization, decreased upon the addition of glucose in *A. nidulans* [29]. Thus, the positive effect of glucose supplementation might be limited to media with high acetate and low glucose concentrations. Supplementing acetate media with glucose probably serves as growth stimulant, leading to a shorter lag phase and faster depletion of the nitrogen source.

### Influence of substrate type and concentration on the side product spectrum

**Fig. 5:**
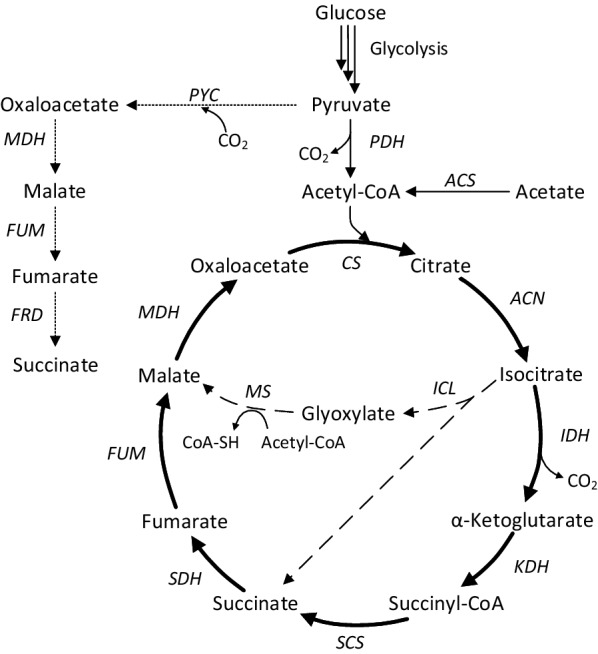
Metabolic pathways in *Aspergillus* species involved in malic acid production. Bold lines represent the TCA cycle, dotted lines the reductive TCA and dashed lines the glyoxylate cycle. Enzymes are abbreviated as follows: *ACN* aconitase, *ACS* acetyl-CoA-synthetase, *CS* citrate synthase, *FRD* fumarate reductase, *FUM* fumarase, *ICL* isocitrate lyase, *IDH* isocitrate dehydrogenase, *KDH* α-ketoglutarate dehydrogenase, *MDH* malate dehydrogenase, *MS* malate synthetase, *PDH* pyruvate dehydrogenase, *PYC* pyruvate carboxylase, *SCS* succinyl-CoA synthetase, *SDH* succinate dehydrogenase

With acetic acid concentrations of 30 g/L and above, malic acid represented the main product and succinic acid the main side product. Compared to the process using glucose as sole carbon source, the malic acid percentage with acetate was decreased in favor of an increased succinic acid production. This observation can be explained by the different production routes with the two substrates. There are three microbial pathways known for malic acid synthesis which are the oxidative tricarboxylic acid cycle (TCA), the reductive TCA (rTCA) and the glyoxylate cycle (Fig. 5). With glucose as carbon source the rTCA was identified as the main pathway leading to malic acid in *Aspergillus* species [30, 31]. This pathway involves the carboxylation of pyruvate originating from glycolysis to oxaloacetate by pyruvate carboxylase and the subsequent production of malate by malate dehydrogenase in the cytosol, resulting in a theoretical yield of 2 mol/mol glucose. Further experiments suggested that the succinic acid produced with glucose as substrate is synthesized via the oxidative TCA only and that the so-produced succinic acid is not the main precursor for the secreted malic acid [30]. With acetate as carbon source, the metabolic situation in *A. oryzae* is less clear. Acetate metabolism generally involves the glyoxylate pathway, a bypass of the oxidative TCA cycle. First, acetate is converted to acetyl-CoA which then enters the TCA cycle where it is used for the synthesis of isocitrate. This transformation involves the action of citrate synthase and aconitase. Isocitrate lyase then catalyzes the conversion of isocitrate to succinate and glyoxylate while malate synthase converts glyoxylate with acetyl-CoA to malate. The existence of the glyoxylate pathway in *A. nidulans* was demonstrated by the induction of the two key enzymes isocitrate lyase and malate synthase in the presence of acetate while no induction was observed when only glucose was present [28, 32]. This finding was further supported by the observation that mutants lacking either of the two enzymes did not grow on acetate [33]. However, it has not been demonstrated whether this is the only pathway leading to the overproduction of malic acid with acetate in *A. oryzae*. The involvement of the glyoxylate pathway in malic acid production from acetate could explain the higher succinic acid yield compared to the process using glucose. Stoichiometrically, one molecule of isocitrate should yield one molecule malate and one molecule succinate. The reason for the lower succinate concentration (with 45 g/L acetic acid: 63 mmol/L malic acid and 45 mmol/L succinic acid) in the results presented here might be caused by a further conversion of succinate to malate by the enzymes succinate dehydrogenase and fumarase. This reveals a possibility for improving malic acid yield and purity. By genetically engineering *A. oryzae* to overexpress succinate dehydrogenase and fumarase, the conversion of succinate to malate could be accelerated, leading to a higher malate accumulation and less by-product formation. In experiments by Brown et al. with engineered *A. oryzae* NRRL 3488 which overexpressed the enzymes pyruvate carboxylase and malate dehydrogenase involved in the reductive TCA cycle as well as a C4-dicarboxylic acid transporter, malic acid yield with glucose as carbon source could be improved by 27% [11].

With an initial concentration of 10 g/L acetic acid no malic acid production was detected, although about 0.6 g/L total acid was produced of which oxalic acid represented the vast majority. Oxalic acid production has been reported in various fungal species and there are two main hypotheses for the reason of its secretion. Oxalic acid is a rather strong acid (pK_A,1_ = 1.23) and therefore might be produced to outcompete other species by medium acidification. This theory is supported by the observation that oxalic acid is mainly accumulated if the pH of the culture broth is at a value of 6 or above [34–36]. Furthermore, oxalic acid acts as efficient chelator for cations which supports fungal growth by mobilizing metal ions [37]. The mechanism of oxalate production with acetate in *A. oryzae* has not been described yet. In *A. niger* with glucose as carbon source, oxalic acid is produced by the conversion of oxaloacetate to oxalate and acetate in the cytosol through oxaloacetate acetylhydrolase [35]. Oxaloacetate is an intermediate of the TCA cycle originating from malate. Other organisms such as the wood-rotting basidiomycete *Gloeophyllum trabeum* feature a second enzyme, glyoxylate dehydrogenase, which is able to synthesize oxalate from glyoxylate in the peroxisome [38]. Why oxalic acid is only produced with low acetate concentrations remains unclear. One could speculate that this is due to the equilibrium of the enzyme reaction catalyzed by oxaloacetate acetylhydrolase. With a high acetate concentration in the medium, the equilibrium is strongly shifted to the side of oxaloacetate while with a low concentration, some of the produced oxaloacetate might react to acetate and oxalate. However, further experiments are required to answer this question.

With glucose as sole carbon source, a noticeable amount of citric acid was secreted which was not the case for cultures with acetate. The cause for this observation might be the different pH development observed with the substrates. The acid spectrum produced by filamentous fungi is strongly influenced by the pH and citric acid is produced most efficiently at low pH values [36]. While during the acid production with glucose, the pH value decreased, it strongly increased when acetate was used as carbon source. Thus, the high pH value in the cultures with acetate probably suppressed citric acid formation.

### Influence of CaCO_3_ addition on malic acid production

During malic acid production using glucose, there are three factors influencing the pH development which are the ammonium consumption, the acid production (both leading to a decrease in pH), and the presence of CaCO_3_. To mitigate against the pH reduction, CaCO_3_ is often added as buffering substance for organic acid production. As the step from pyruvate to oxaloacetate involves CO_2_ fixation, it is assumed that CaCO_3_ furthermore functions as CO_2_ supply [16, 39]. The third reason for adding CaCO_3_ is the formation of calcium malate precipitates, allowing for the production of higher malic acid concentrations. The utilization of acetate, however, adds another factor which influences the pH development. Upon acetate consumption, the medium pH strongly increases which might inhibit the metabolic performance of *A. oryzae*. This observation posed the question whether the presence of CaCO_3_ is necessary for malic acid production using acetate. The results presented here show that high concentrations of 70–90 g/L CaCO_3_ are required for efficient malic acid production. In the absence of CaCO_3_, less than 1 g/L malic acid was produced, clearly indicating the necessity for CaCO_3_ in the medium. In acetate media, CaCO_3_ does not contribute to the pH buffering in the manner it does for media with glucose as the pH increases. Furthermore, if malate is not produced with the involvement of the enzyme pyruvate carboxylase which fixes CO_2_, CaCO_3_ neither fulfills the role of a CO_2_-donor. This could lead to the conclusion that the main function of CaCO_3_ is the formation of calcium malate. However, the results presented here cannot answer this question conclusively. Evaluating the influence of different carbonates such as Na_2_CO_3_ or K_2_CO_3_ could be interesting as these are soluble carbonates which can be handled more easily than the poorly soluble CaCO_3_.

### Considerations regarding the utilization of acetate derived from lignocellulose

The results presented in this work show that for an efficient biomass production with *A. oryzae*, acetic acid concentrations in the range of 40–55 g/L are optimal while regarding the acid production 45–50 g/L are ideal. In the context of bioeconomy, the utilization of acetate-containing waste or side streams derived from lignocellulose is targeted. Acetic acid is for example contained in lignocellulosic hydrolysates, in side streams of fast pyrolysis or produced during syngas fermentation. The acetic acid content in these products encompasses a broad range. In lignocellulosic hydrolysates the acetic acid content depends on the degree of acetylation of the biomass or the amount of hemicellulose, respectively. Usually, the acetic acid concentration in lignocellulosic hydrolysates is below 15 g/L [40–42]. According to the results presented in this work, these concentrations are too low for malic acid production with *A. oryzae*, however, they can be used for biomass production. Within an integrated bioeconomy, it is also conceivable that the sugars in these hydrolysates are first used in other microbial processes and the remaining acetate then is used for biomass production with *Aspergillus*. Higher acetate concentrations are contained in pyrolysis products or in the culture broth after syngas fermentation. During fast pyrolysis, biomass is subjected to high temperatures for a few seconds while oxygen is absent. The remaining vapors are condensed subsequently and yield a bio-oil and, in some processes using ash-rich biomasses, an aqueous condensate. The acetic acid concentration in these condensates often ranges between 45 and 80 g/L acetic acid, which is a suitable range for malic acid production with *A. oryzae* [3, 5]. Through further processing of fast pyrolysis products, biomass-derived syngas is obtained. With acetogenic microorganisms this gas, mainly containing CO, CO_2_ and H_2_, can be converted to acetic acid. With *Clostridium ljungdahlii* acetic acid concentrations of about 21 g/L were reported [4] while with *Acetobacterium woodii* titers of over 50 g/L were measured [43, 44]. Malic acid production with acetate obtained from *C. ljungdahlii* has already been reported by Oswald et al. [15]. During their fermentations, 14–18 g/L acetic acid was produced and only low malic acid concentrations of 0–1.8 g/L were measured. As demonstrated by the results obtained here, the acetic acid concentrations produced during the syngas fermentation of Oswald et al. was just on the merge of the malate production limit. Therefore, choosing a different acetogenic microorganism which is capable of a higher acetate production is advisable. However, compared to directly using pyrolysis products like the bio-oil or the aqueous condensate, another fermentation step is required. In this sense, the direct utilization of the pyrolytic aqueous condensate for malic acid production could be especially interesting as this is a low-value side stream which, due to its low energy content, can only be partially utilized in the subsequent gasification process [45]. Therefore, further exploring this possibility is highly interesting in the context of a bio-based economy.

## Conclusions

In this work, new insights regarding the utilization of acetate as substrate for growth and malic acid production with *A. oryzae* are provided. While for biomass formation acetic acid concentrations of 40–55 g/L were best suited, the highest malic acid titers were observed with a narrower substrate concentration range of 45–50 g/L. Considering that the time point of maximum productivity was lower for cultures with 45 g/L acetic acid and no substrate remained unused, this concentration was identified as the optimum for malic acid production. A comparison of the side product spectrum demonstrated a dependency of the produced acids on the initial acetate concentration. While oxalic acid represented the main product for low concentrations of 10–20 g/L acetic acid, this place was taken by malic acid for substrate concentrations of 30 g/L and above. The identification of succinic acid as a major side product could help to increase malic acid productivity by reinforcing the metabolic pathway leading from succinic to malic acid through metabolic engineering. By the addition of low glucose amounts to acetate media the acid yield was increased, and the production process was accelerated. Furthermore, it was demonstrated that adding CaCO_3_, a neutralizing agent commonly applied in malic acid production with glucose, is also crucial for acid production using acetate, although the mechanism of action could not be determined conclusively. Performing malic acid production as fed-batch process with feeding of acetic acid to keep both pH and substrate concentration in a stable range could be interesting for future studies to improve malic acid yields.

## Methods

### Microorganism and culture conditions

All chemicals were purchased from Sigma-Aldrich (Germany) or Carl-Roth (Germany). *Aspergillus oryzae* DSM 1863 was obtained from DSMZ strain collection (Deutsche Sammlung von Mikroorganismen und Zellkulturen GmbH, Braunschweig, Germany).

#### Spore collection

For spore collection *A. oryzae* was grown on a minimal medium for *Aspergillus* species containing 6 g/L NaNO_3_, 0.52 g/L KCl, 0.52 g/L MgSO_4_·7H_2_O, and 1.52 g/L KH_2_PO_4_ [46]. The pH was set to 6.5 with NaOH. 15 g/L glucose monohydrate, 2 mL/L of 1000 × Hutner’s trace elements, and 15 g/L agar were added afterwards and the medium was sterilized for 20 min at 121 °C. 1000 × Hutner’s trace element solution is composed of 5 g/L FeSO_4_·7H_2_O, 50 g/L EDTA-Na_2_, 22 g/L ZnSO_4_·7H_2_O, 11 g/L H_3_BO_3_, 5 g/L MnCl_2_·4H_2_O, 1.6 g/L CoCl_2_·6H_2_O, 1.6 g/L CuSO_4_·5H_2_O, and 1.1 g/L (NH_4_)_6_Mo_7_O_24_·4H_2_O, pH 6.5 [47]. Conidia were harvested with 50% glycerol after 7–10 days of incubation at 30 °C by gently scraping the plate surface with a sterile inoculation loop. The conidia suspension was filtered through Miracloth (Merck KGaA, Darmstadt, Germany) and diluted to a concentration of 3 × 10^7^ conidia per mL with 50% glycerol. Aliquots were stored at − 80 °C.

#### Pre-culture for biomass formation

Malic acid production was performed as a two-stage process consisting of a pre-culture for biomass production and a main culture for acid production. In the pre-culture an excess of nitrogen was present to promote biomass formation while the nitrogen supply in the main culture was reduced to favor acid production over cell growth. The pre-culture medium consisted of 5–70 g/L sodium hydroxide-neutralized acetic acid (pH 6.5) or 40 g/L glucose monohydrate as carbon source, 4 g/L (NH_4_)_2_SO_4_, 0.75 g/L KH_2_PO_4_, 0.98 g/L K_2_HPO_4_, 0.1 g/L MgSO_4_·7H_2_O, 0.1 g/L CaCl_2_·2H_2_O, 5 mg/L NaCl, and 5 mg/L FeSO_4_·7H_2_O. While for the glucose medium the pH was not adjusted, it was adjusted to 6.5 for the pre-culture media containing acetate. All pre-culture media were sterilized by autoclaving for 20 min at 121 °C. For biomass formation, 100 mL of pre-culture medium in 500-mL baffled Erlenmeyer shake flasks was inoculated with 3 × 10^7^ conidia and incubated for 48 h (acetate media) or 24 h (glucose media) at 30 °C and 100 rpm. After incubation, the biomass used for the inoculation of the main culture was separated from the pre-culture medium by filtration through Miracloth (Merck) and washed with double-distilled water. For the growth experiments in the pre-culture, samples of 1.9 mL were taken in the beginning of the experiment and after 48 h to determine substrate and ammonium consumption. Afterwards, the entire content of a flask was used for biomass determination via cell dry weight. All experiments were performed as biological triplicates.

#### Main culture for organic acid production

The main culture medium contained 10–55 g/L sodium hydroxide-neutralized acetic acid (pH 5.5) or 120 g/L glucose monohydrate as carbon source, 1.2 g/L (NH_4_)_2_SO_4_, 0.1 g/L KH_2_PO_4_, 0.17 g/L K_2_HPO_4_, 0.1 g/L MgSO_4_·7H_2_O, 0.1 g/L CaCl_2_·2H_2_O, 5 mg/L NaCl, and 60 mg/L FeSO_4_·7H_2_O. Additionally, 90 g/L CaCO_3_ was added to the main culture media unless stated otherwise. While for the glucose medium the pH was not adjusted, it was adjusted to 5.5 for the main culture media containing acetate. The pH of the main culture media was set to a lower pH than the pre-culture media to moderate the pH increase caused by the CaCO_3_ addition. For main cultures without CaCO_3_, the pH was set to 6.50. All media were sterilized by autoclaving for 20 min at 121 °C. For the media containing 45 g/L acetic acid and 5–15 g/L glucose, the glucose solution was sterile filtered and added to the medium after autoclaving. For the experiments with 45 g/L acetic acid + 5–15 g/L glucose in the main culture medium, the pre-culture was grown in medium containing 45 g/L acetic acid. For acid production, 100 mL of the main culture medium was transferred to 500-mL baffled Erlenmeyer shake flasks, inoculated with 0.75 g of the washed biomass obtained during the pre-culture and incubated at 120 rpm and 32 °C. Samples of 4 mL were taken at the indicated time points for pH, organic acid and ammonium measurement. All experiments per performed as biological triplicates.

### Analytics

#### Characterization and quantification of biomass formation in the pre-culture

Fungal morphology was characterized by light microscopy using a Nikon Eclipse E200 equipped with a DFK 23U274 camera (Imaging Source, Bremen, Germany) supported by the software NIS-Elements D ver. 4.50.

For quantification of biomass formation in the pre-culture, the dry biomass weight was determined. For this purpose, the culture broth of an entire flask was filtered through pre-dried and pre-weighed Miracloth (Merck) after an incubation time of 48 h and washed thoroughly with double-distilled water. The biomass, together with the Miracloth, was dried at 70 °C for at least 48 h until weight constancy. The weight of the biomass-containing Miracloth was measured with precision scales and used for the calculation of the dry biomass weight in g/L.

#### Organic acid quantification

Organic acids were quantified by HPLC. Samples were pretreated with sulfuric acid to release precipitated calcium malate as described by Ochsenreither et al. [10]. A sample volume of 1 mL was mixed with 3 mL of water and 1 mL of 3 M H_2_SO_4_, followed by an incubation period of 20 min at 80 °C. Subsequently, 1 mL of the suspension was centrifuged for 10 min at 20,000x*g* and the supernatant was used for HPLC analysis. The analysis was performed using a standard HPLC device (Agilent 1100 Series, Agilent, Germany) equipped with a Rezex ROA organic acid H + (8%) column (300 by 7.8 mm, 8 m; Phenomenex) and a Rezex ROA organic acid H + (8%) guard column (50 by 7.8 mm). Samples were analyzed at 60 °C with 3 mM H_2_SO_4_ as mobile phase and a flow of 0.5 mL/min. The injection volume was 10 µL and detection was performed with a UV detector at 220 nm.

#### Glucose quantification

For glucose determination by HPLC, 1 mL of the culture broth was centrifuged for 10 min at 20,000x*g* and the supernatant was used for analysis using the same column set-up as for organic acid determination. Samples were analyzed at 50 °C with 5 mM H_2_SO_4_ and a flow of 0.5 mL/min. The injection volume was 10 µL and glucose was detected with a refractive index detector.

#### Ammonium quantification

Ammonium concentration was quantified photometrically using the Spectroquant kit (114752, Merck KGaA, Darmstadt, Germany). The assay was scaled down to a volume of 200 µL and sample supernatants were measured in duplicate in microtiter plates according to the manufacturer’s instructions.

### Data analysis

Product formation and substrate consumption for acetate and glucose fermentation experiments were fitted using a four-parameter logistic equation in a data analysis and graphing software (OriginPro 2020, OriginLab Corporation, Northampton, USA). As iteration algorithm the Levenberg–Marquardt algorithm was used. The used equation was the following:

1 $$y\left(x\right)= \frac{{A}_{1}- {A}_{2}}{1+{(\frac{x}{{x}_{0}})}^{p}}+{A}_{2}.$$

In Eq. (1), A_1_ indicates the initial carbon source or product concentration, A_2_ indicates the final carbon source or product concentration, x_0_ indicates the process time when half of the amount of carbon source is consumed or half of the maximum product concentration is reached, and p is a shape parameter.

Product formation and substrate consumption for mixed substrate fermentation was fitted using a modified Gompertz model [48]:

2 $$y\left(x\right)=A \mathrm{exp}(-\mathrm{exp}\left(\frac{ve}{A}\left(d-x\right)+1\right),$$

with y being the carbon source or product concentration, A the upper asymptote value, v the acid formation or product consumption rate and d the lag phase duration. For both equations, the first derivative of the fitted curve was used for the calculation of the maximum production rates and corresponding time points.

## Supplementary Information

**Additional file 1: Figure S1.** Evaluation of the optimum initial pH for malic acid production with acetate. Initial medium pH-values of 4.5, 5.0, 5.5, 6.0 and 6.5 were tested and malic acid concentration after 192 h is displayed. Experiments were performed with 45 g/L acetic acid, 1.2 g/L (NH4)2SO4 and 90 g/L CaCO3. Cultures were incubated at 32 °C and 120 rpm. Data points are the mean of biological triplicates and error bars indicate the standard deviation. ** Figure S2.** Determination of ammonium consumption with different substrate concentrations. Cultures were incubated at 32 °C and 120 rpm in the presence of 90 g/L CaCO3. Data points are the mean of biological triplicates and error bars indicate the standard deviation. HAc acetic acid, Glc glucose. ** Figure S3.** pH-values depending on substrate type and concentration. Cultures were incubated at 32 °C and 120 rpm in the presence of 90 g/L CaCO3. Data points are the mean of biological triplicates and error bars indicate the standard deviation. HAc acetic acid, Glc glucose. **Figure S4.**pH-values depending on the CaCO3 concentration. Cultures were incubated at 32 °C and 120 rpm with 45 g/L acetic acid. Data points are the mean of biological triplicates and error bars indicate the standard deviation. ** Figure S5.** Determination of ammonium consumption depending on the CaCO3 concentration. Cultures were incubated at 32 °C and 120 rpm with 45 g/L acetic acid. Data points are the mean of biological triplicates and error bars indicate the standard deviation.

## Data Availability

The datasets used and analyzed during the current study are available from the corresponding author on reasonable request.
