# Supplementary material for: Acetate as substrate for l-malic acid production with Aspergillus oryzae DSM 1863
Source: Biotechnol Biofuels. 2021 Feb 23;14:48. doi: 10.1186/s13068-021-01901-5 (PMC7903783; doi:10.1186/s13068-021-01901-5)
Supplement: Supplementary file 1 — Additional file 1: Figure S1. Evaluation of the optimum initial pH for malic acid production with acetate. Initial medium pH-values of 4.5, 5.0, 5.5, 6.0 and 6.5 were tested and malic acid concentration after 192 h is displayed. Experiments were performed with 45 g/L acetic acid, 1.2 g/L (NH4)2SO4 and 90 g/L CaCO3. Cultures were incubated at 32 °C and 120 rpm. Data points are the mean of biological triplicates and error bars indicate the standard deviation. Figure S2. Determination of ammonium consumption with different substrate concentrations. Cultures were incubated at 32 °C and 120 rpm in the presence of 90 g/L CaCO3. Data points are the mean of biological triplicates and error bars indicate the standard deviation. HAc acetic acid, Glc glucose. Figure S3. pH-values depending on substrate type and concentration. Cultures were incubated at 32 °C and 120 rpm in the presence of 90 g/L CaCO3. Data points are the mean of biological triplicates and error bars indicate the standard deviation. HAc acetic acid, Glc glucose. Figure S4.pH-values depending on the CaCO3 concentration. Cultures were incubated at 32 °C and 120 rpm with 45 g/L acetic acid. Data points are the mean of biological triplicates and error bars indicate the standard deviation. Figure S5. Determination of ammonium consumption depending on the CaCO3 concentration. Cultures were incubated at 32 °C and 120 rpm with 45 g/L acetic acid. Data points are the mean of biological triplicates and error bars indicate the standard deviation. [file 13068_2021_1901_MOESM1_ESM.pdf]

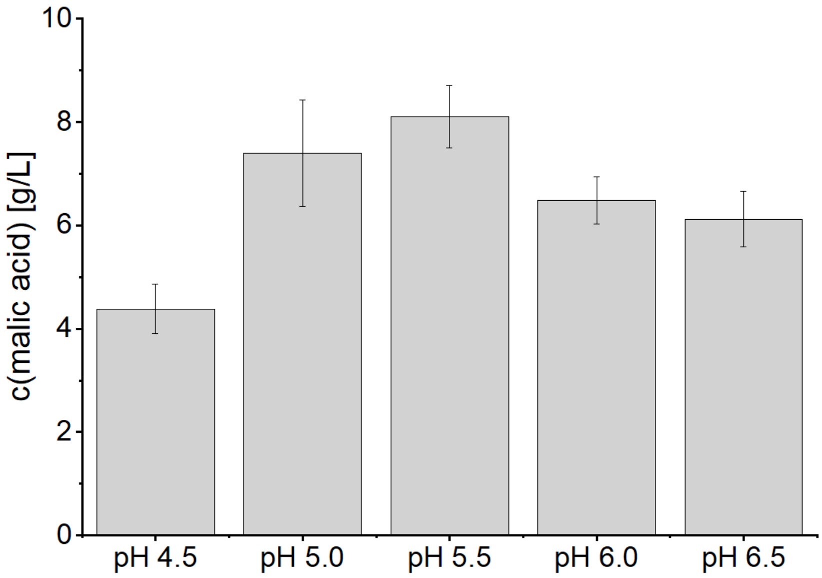

Figure S1: Evaluation of optimum initial pH for malic acid production with acetate. Initial medium pH-values of 4.5, 5.0, 5.5, 6.0 and 6.5 were tested and malic acid concentration after 192 h is displayed. Experiments were performed with 45 g/L acetic acid, 1.2 g/L $(NH_4)_2SO_4$ and 90 g/L $CaCO_3$. Cultures were incubated at 32 °C and 120 rpm. Data points are the mean of biological triplicates and error bars indicate the standard deviation.

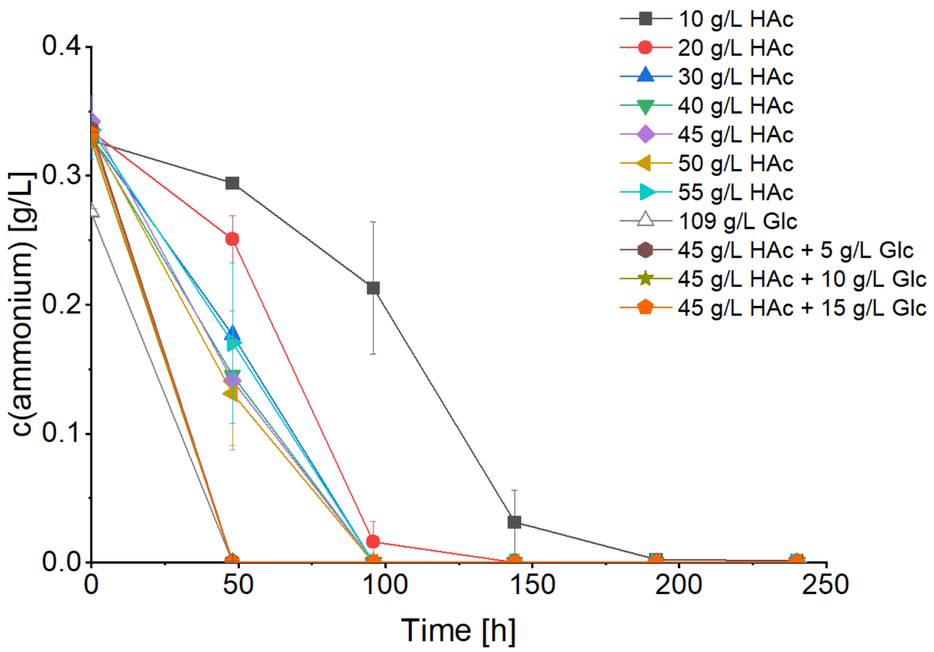

Figure S2: Determination of ammonium consumption with different substrate concentrations. Cultures were incubated at 32 °C and 120 rpm in the presence of 90 g/L $CaCO_3$. Data points are the mean of biological triplicates and error bars indicate the standard deviation. HAc = acetic acid, Glc = glucose.

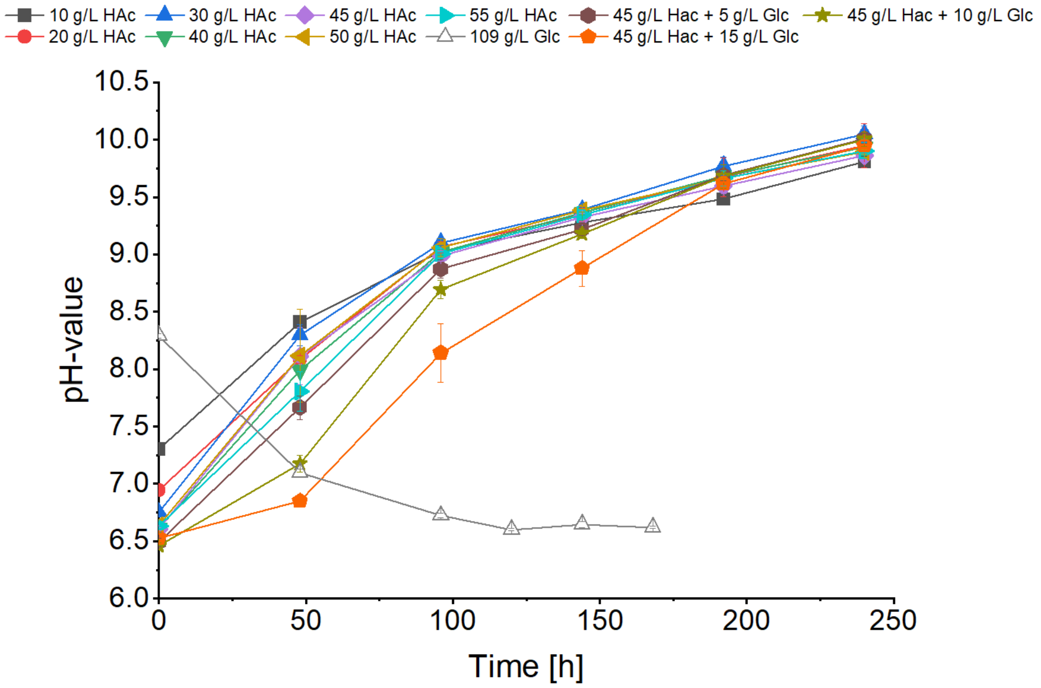

Figure S3: pH-values depending on substrate type and concentration. Cultures were incubated at 32 °C and 120 rpm in the presence of 90 g/L $CaCO_3$. Data points are the mean of biological triplicates and error bars indicate the standard deviation. HAc = acetic acid, Glc = glucose.

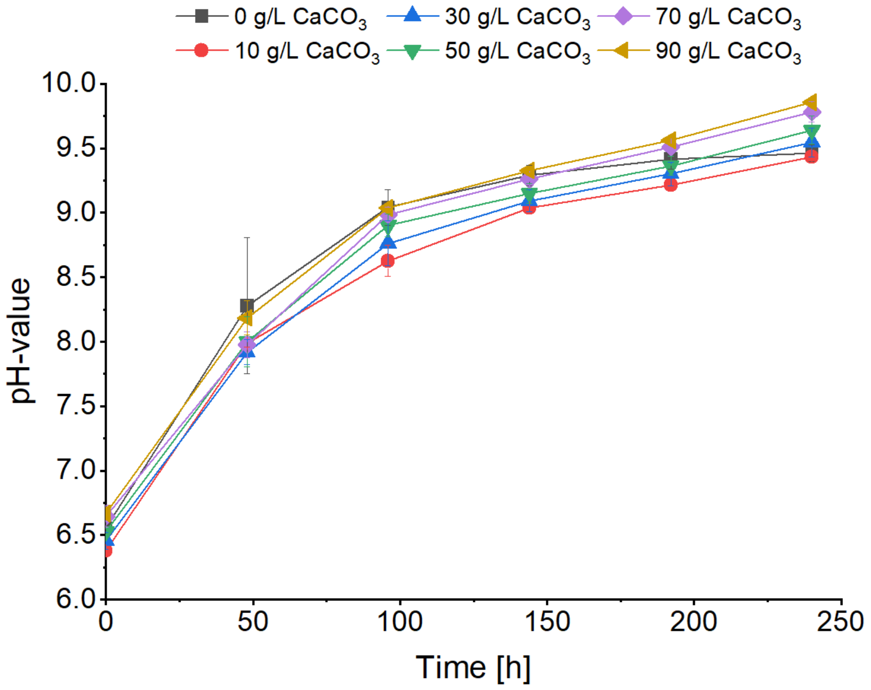

Figure S4: pH-values depending on the $CaCO_3$ concentration. Cultures were incubated at 32 °C and 120 rpm with 45 g/L acetic acid. Data points are the mean of biological triplicates and error bars indicate the standard deviation.

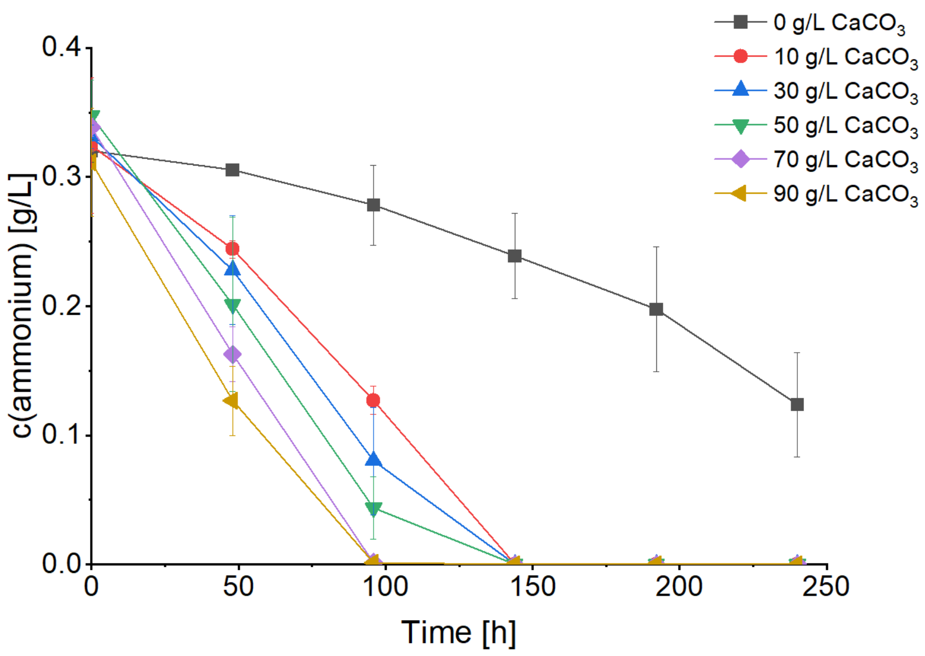

Figure S5: Determination of ammonium consumption depending on the $CaCO_3$ concentration. Cultures were incubated at 32 °C and 120 rpm with 45 g/L acetic acid. Data points are the mean of biological triplicates and error bars indicate the standard deviation.
